# Multiple Trigger Points for Quantifying Heat-Health Impacts: New Evidence from a Hot Climate

**DOI:** 10.1289/ehp.1409119

**Published:** 2015-07-28

**Authors:** Diana B. Petitti, David M. Hondula, Shuo Yang, Sharon L. Harlan, Gerardo Chowell

**Affiliations:** 1 Department of Biomedical Informatics, and; 2 Department of Family, Community and Preventive Medicine, College of Medicine-Phoenix, University of Arizona, Phoenix, Arizona, USA; 3 Center for Policy Informatics, Arizona State University, Phoenix, Arizona, USA; 4 School of Geographical Sciences and Urban Planning, Arizona State University, Tempe, Arizona, USA; 5 School of Human Evolution & Social Change, Arizona State University, Tempe, Arizona, USA; 6 School of Public Health, Georgia State University, Atlanta, Georgia, USA

## Abstract

**Background:**

Extreme heat is a public health challenge. The scarcity of directly comparable studies on the association of heat with morbidity and mortality and the inconsistent identification of threshold temperatures for severe impacts hampers the development of comprehensive strategies aimed at reducing adverse heat-health events.

**Objectives:**

This quantitative study was designed to link temperature with mortality and morbidity events in Maricopa County, Arizona, USA, with a focus on the summer season.

**Methods:**

Using Poisson regression models that controlled for temporal confounders, we assessed daily temperature–health associations for a suite of mortality and morbidity events, diagnoses, and temperature metrics. Minimum risk temperatures, increasing risk temperatures, and excess risk temperatures were statistically identified to represent different “trigger points” at which heat-health intervention measures might be activated.

**Results:**

We found significant and consistent associations of high environmental temperature with all-cause mortality, cardiovascular mortality, heat-related mortality, and mortality resulting from conditions that are consequences of heat and dehydration. Hospitalizations and emergency department visits due to heat-related conditions and conditions associated with consequences of heat and dehydration were also strongly associated with high temperatures, and there were several times more of those events than there were deaths. For each temperature metric, we observed large contrasts in trigger points (up to 22°C) across multiple health events and diagnoses.

**Conclusion:**

Consideration of multiple health events and diagnoses together with a comprehensive approach to identifying threshold temperatures revealed large differences in trigger points for possible interventions related to heat. Providing an array of heat trigger points applicable for different end-users may improve the public health response to a problem that is projected to worsen in the coming decades.

**Citation:**

Petitti DB, Hondula DM, Yang S, Harlan SL, Chowell G. 2016. Multiple trigger points for quantifying heat-health impacts: new evidence from a hot climate. Environ Health Perspect 124:176–183; http://dx.doi.org/10.1289/ehp.1409119

## Introduction

Many studies have retrospectively examined high environmental temperature and mortality. This research has largely focused on estimating excess deaths from all-cause mortality and on the statistical identification of a single threshold temperature above which deaths increase (e.g., Hajat and Kosatsky 2010; McMichael et al. 2008). Importantly, the temperature thresholds identified in such studies have been proposed as a basis for the activation of heat-health warning systems and other public health interventions (e.g., Henderson and Kosatsky 2012; Pascal et al. 2006). Other applications of retrospective analyses include assessment of the potential future health effects of local-, regional-, or global-scale climate change (e.g., Huang et al. 2011).

A related and rapidly accumulating body of research assesses the relationship between high temperature and health events other than mortality: hospital admissions and emergency department (ED) visits (Hess et al. 2014; reviews by Kravchenko et al. 2013; Martiello and Giacchi 2010; Ye et al. 2012), hospital admissions among patients seen in the ED (Pillai et al. 2014), ambulance/emergency response calls (Alessandrini et al. 2011; Hartz et al. 2013; Nitschke et al. 2011; Schaffer et al. 2012; Williams et al. 2012a, 2012b), teleradiology calls (Brunetti et al. 2014), and outpatient visits (Pudpong and Hajat 2011). However, only a few studies have considered more than one measure of health effects associated with heat, for a single geographic region, at the same time (e.g., Kovats et al. 2004; Williams et al. 2012a, 2012b).

The fact that extreme heat persists as a public health challenge (Berko et al. 2014) despite compelling evidence for its adverse effects on health calls for new approaches toward preparedness and intervention strategies. Here, we propose that it is possible to better understand and mitigate the current and future risks posed by high temperatures with adaptation strategies based on comprehensive and contextualized weather information spanning a range of health outcomes associated directly and indirectly with heat.

Opportunities for improving public health strategies aimed at mitigating the effects of heat on health may lie at the intersection of many of the ideas and methodologies that have been brought forward to date. For example, functional forms of heat-health relationships are dependent on the local setting (Anderson and Bell 2009; Curriero et al. 2002). In addition, the relationship between temperature and mortality and morbidity may have different functional forms within a given location (Kovats et al. 2004). Intervention strategies aimed at particular populations (e.g., outdoor workers vs. elderly residents) would be most effective if they considered the diagnosis and severity of health events that are most relevant for that population. Furthermore, various definitions of temperature thresholds are employed in the literature, some of which are brought forward with little more than generalities about the purpose of identifying such metrics. The suite of different conceptualizations of “thresholds” for heat-related health effects proposed thus far (e.g., Davis et al. 2003; Li et al. 2013; Pascal et al. 2006) offers considerable variability in terms of utility for heat-health adaptation strategies.

The aim of this study was to systematically identify the meteorological conditions under which there might be reasons to enact heat-health interventions based on empirical relationships between hot weather and illness or death. Our concern was that an opportunity to mitigate a large portion of adverse health outcomes associated with heat may be lost if the activation of preventive measures for heat-related illness and death is keyed to temperatures at which all-cause mortality statistically exceeds a seasonal baseline. In hot climates such as the one that characterizes Maricopa County, Arizona, health events associated with heat exposure may begin to occur well before a statistical threshold temperature for all-cause mortality is crossed (Harlan et al. 2014). Moreover, there are a suite of health events and diagnoses associated with heat that may respond differently to ambient conditions. Hence, our approach moves beyond the use of a single threshold by considering multiple different temperatures (henceforth referred to as trigger points) to characterize the complex relationship between heat and health.

## Materials and Methods

*Study setting.* The study setting, Maricopa County, Arizona, USA, (2012 population, 3.9 million) comprises the city of Phoenix (2012 population 1.5 million), eight other contiguous cities with populations ranging from 100,000 to 400,000, 15 adjoining municipalities, and three Native American communities. In Phoenix, the daily mean temperature in the summer (June–September), 33^o^C (91.4^o^F), is the highest of all major United States metropolitan areas [National Oceanic and Atmospheric Administration (NOAA) 2013]. In the Phoenix metropolitan area, 95% of occupied housing units have central air conditioning, which is > 50% greater than the national average [American Housing Survey (AHS) 2014].

*Health data.* The study considered 10 different health events: all-cause mortality; cardiovascular (CVD) mortality, hospitalizations, and emergency department (ED) visits; heat-related deaths, hospitalizations, and ED visits; and mortality, hospitalizations, and ED visits for conditions that are consequences of heat and dehydration. The selected events represent different levels of severity for personal suffering and loss (death, hospitalization, emergency treatment) and health problems that represent different types of risk profiles: all-cause mortality (broadest scope, most often studied), CVD (underlying disease, greater physiological susceptibility, large affected population), and direct heat exposure (acute, specific, situational).

We obtained mortality data for 1 January 2000–31 December 2011 from the Arizona Department of Health Services (ADHS). Each record included date of death, underlying cause of death coded using the World Health Organization’s (WHO’s) *International Classification of Diseases, 10th Revision* (ICD-10), and text entered in the contributing causes of death fields on the death certificate.

We also obtained data on hospitalizations and ED visits at facilities located in Maricopa County for 1 January 2008–31 December 2012 from ADHS. All Arizona hospitals except Veteran’s Administration, military, Indian Health Services, and behavioral health hospitals were required by law to report information to ADHS during this period. Information obtained included admission and discharge dates in addition to discharge diagnoses and causes of injury coded using the WHO’s *International Classification of Diseases, 9th Revision, Clinical Modification* (ICD-9-CM). During the study period, codes were captured on ≤ 25 discharge diagnoses and ≤ 9 external causes of injury for each individual for both hospitalizations and ED visits.

In our analysis based on all-cause mortality, we excluded most external causes of death. Following the method reported by Harlan et al. (2014), we excluded ICD-10 codes S00–99, T00–66, T68–98, U00–99, X00–29, 32, 33–53, 55–84, Y00–98, and Z00–99 but included T67.x, X30, X32, and X54 because these are heat-related. The conditions used to define mortality and morbidity events in the CVD category and their corresponding ICD-10 and ICD-9 codes are listed in Supplemental Material, Table S1. We conducted two separate analyses of CVD hospitalization and ED visits, one using only the first discharge diagnosis code to define a patient as having a CVD event and one using all (≤ 25) discharge diagnosis codes to define a patient as having a CVD event. Only data for CVD as the first discharge diagnosis are discussed because the results were essentially the same when CVD as any discharge diagnosis was examined (data not shown).

The conditions used to define a mortality or morbidity event as heat-related and the corresponding ICD-10 and ICD-9-CM codes are listed in Supplemental Material, Table S2. In the heat-related mortality category, terms associated with exposure to high environmental heat (e.g., “heat exhaustion”) entered as free text in the underlying cause-of-death fields of the death certificate Part 1 were also used to define a death as heat-related (see Supplemental Material, Table S2). Hospitalizations and ED visits were classified as directly heat-related if any discharge diagnosis code (≤ 25 possible for any individual hospitalization or ED visit) or external cause of injury code (≤ 9 possible) corresponded to the predefined ICD codes for this category.

A category of conditions that are possible consequences of heat and/or dehydration was defined based on a model of the physiologic and pathophysiologic effects of heat. The Supplemental Material presents a graphic depiction of the model (see Supplemental Material, Figure S1) along with a list of the ICD-10 and ICD-9 codes for this category (see Supplemental Material, Table S3). Hospitalizations and ED visits were classified as possible consequences of heat and/or dehydration if any of the ≤ 25 discharge diagnosis codes or ≤ 9 external cause of injury codes corresponded to the predefined ICD-9 codes for this category.

Individuals who were hospitalized more than once or who had more than one ED visit were counted multiple times. However, individuals admitted to the hospital who were also seen in the ED for that same episode of illness were counted only once, as a hospitalization. Fatal outcomes during or after being hospitalized or in the ED or after being seen in the ED were counted in both the mortality analysis and the analyses of hospitalization and ED visits because the available data did not permit deduplication across data sources.

*Ethics review.* The study was reviewed and approved by both the Arizona State University Institutional Review Board and the ADHS Human Subjects Review Board.

*Meteorological data.* We obtained hourly air temperature and relative humidity data from the National Weather Service (NWS) monitoring station at Sky Harbor International Airport in Phoenix for the period 1 January 2000–31 December 2012. From these data, we calculated six temperature metrics: daily minimum, mean, and maximum air temperature (T_min_, T_mean_, and T_max_, respectively) and daily minimum, mean, and maximum heat index (HI_min_, HI_mean_, HI_max_, respectively). We used the lowest and highest daily values for the minimum and maximum, respectively, and the average of 24-hr temperatures as the daily mean. The HI estimates thermal stress resulting from ambient conditions by combining temperature and humidity into a single variable. Here, we used an NWS HI algorithm that parameterizes the Steadman apparent temperature model (NWS 2014; Steadman 1979). Detail is provided in the Supplemental Material, “Algorithm for Calculation of Heat Index Based on Steadman 1979; NWS 2014.”

*Analysis.* To minimize the effect of season on health, we restricted the analysis to the period 15 May–15 October of each year. In this setting, we found same-day and 1-day lag temperature and HI to be among the most important discriminators between days with high and low mortality, hospitalizations, or ED visits. Thus, these variables were deemed to have stronger associations with health events than were other possible variables (e.g., dew point temperature, departures from climatological normals, variables with longer lags or smoothers including conceptualizations of “heat waves”). A full examination of this larger suite of potential explanatory variables is outside the scope of this analysis, but the six variables we chose to examine are in line with those found to be most relevant to health (e.g., Anderson and Bell 2009; Hajat et al. 2006).

We estimated the relationship between the temperature metrics and the health events using a generalized additive model (GAM) (Hastie and Tibshirani 1990). Separate models were constructed for each of the six temperature metrics and for each of the 10 different types of health events considered. For the CVD category, we used a 1-day lag between the air temperature or HI metric and the events [following the method of Harlan et al. (2014)]. For the other event types, we examined same-day effects.

For all-cause mortality and CVD events (mortality, hospitalization, and ED visits), the GAM took the form:

*log(M) = month + year + s(env, k = 4)*, [1]

where *M* is a time series of mortality or CVD morbidity, *month* is a factor term representing month of year, *year* is a factor term representing calendar year, *s* is a fixed thin-plate regression spline with k-1 degrees of freedom, and *env* represents any of the six temperature metrics considered.

Because the study was restricted to warmer months (15 May–15 October), we did not combine seasonal and long-term trend effects into one single temporal variable (e.g., Anderson and Bell 2009; Hondula et al. 2013). Restricting the analysis to the mid-May to mid-October window greatly reduced concerns regarding confounding effects from annual variability in all-cause and CVD event rates, which are accounted for by the *month* term in Equation 1. We found that replacing *month* with a higher-resolution time variable such as day of year had no appreciable influence on the overall results (data not shown). The models for heat-related events did not include the term *month* because any seasonality in these events was believed to be directly related to temperature.

Based on the modeled relationships between each of the six temperature metrics and the 10 health events, we calculated three separate trigger points to compare the relative sensitivity to hot weather across metrics and events. We defined trigger points as temperatures at which there is a prespecified increase in the occurrence of the given health event. The minimum risk temperature (MRT) is conceptually similar to the temperature of minimum mortality described by Curriero et al. (2002), Keatinge et al. (2000), and Kinney et al. (2008). For health events that would not be expected in the absence of high temperatures (heat-related mortality, hospitalizations, and ED visits and events associated with mortality, hospitalization, and ED visits that were categorized as consequences of heat and dehydration), we defined the MRT as the temperature at which the fewest events were observed (which was typically the lowest temperature at which an event was observed). For health events that may be influenced by, but are not entirely dependent on, high temperature (all-cause mortality and CVD events), we defined the MRT as the lowest temperature above which a consistent increase in relative risk was observed (i.e., the slope of the temperature–health event relationship is always positive above the MRT).

The increasing risk temperature (IRT) was defined as the lowest temperature at which the relative risk of a given health event was greater than the upper 95% confidence limit of the MRT. Thus, the IRT is an indicator of the lowest temperature at which there is a larger impact on the health event than what is expected under optimal weather conditions.

The excess risk temperature (ERT) was defined as the lowest temperature above the MRT at which the relative risk of a particular health event was statistically significantly greater than 1.0 based on the lower bound of the 95% confidence interval for the relative risk above 1.0. The reference level for estimation of relative risk is the expected rate of the health event in a given month. Conceptually, the ERT is the lowest temperature at which mortality or morbidity rates are modeled to be anomalously greater than the number of events expected based on normal summer weather and, for some of the health events considered, other temporal factors that drive seasonal variability in the time series of event counts.

MRTs, IRTs, and ERTs could be undefined.

The sensitivity of the results to the time period of record was assessed by replicating the abovementioned procedure for several different combinations of study period start and end years.

## Results

During the time period for which both mortality and morbidity data were available, the number of morbidity events greatly exceeded the number of mortality events (Table 1). The average number of heat-related deaths per year for the months in the analysis from 2008 to 2011 (*n* = 35) was 10.1% of the average number of heat-related hospitalizations (*n* = 346), which in turn was 25.4% of the average number of heat-related ED visits (*n* = 1,361). For reference, in Maricopa County during the period 2008–2011, approximately 460,000 hospitalizations and 1.1 million ED visits (not admitted to the hospital) per year were recorded [Agency for Healthcare Research and Quality (AHRQ) 2014].

**Table 1:** For categories and types of events, total and average events per year for months in analysis.

Category/event type	Years in analysis	Total number of events for months in analysis	Average events per year for months in analysis
All-cause mortality		112,853	9,404
Cardiovascular
Mortality	2000–2011	30,531	2,544
Hospitalization^*a*^	2008–2012	32,614	6,523
ED visit^*a*^	2008–2012	6,831	1,366
Heat-related
Mortality	2000–2011	424	35
Hospitalization	2008–2012	1,731	346
ED visit	2008–2012	6,803	1,361
Consequences of heat and dehydration
Mortality	2000–2011	1,458	122
Hospitalization	2008–2012	357,363	71,473
ED visit	2008–2012	233,636	46,727
ICD-9 and ICD-10 codes used to define categories of conditions are given in the Supplemental Material, Tables S1–S3. ^***a***^First discharge diagnosis only.			

**Figure 1:**
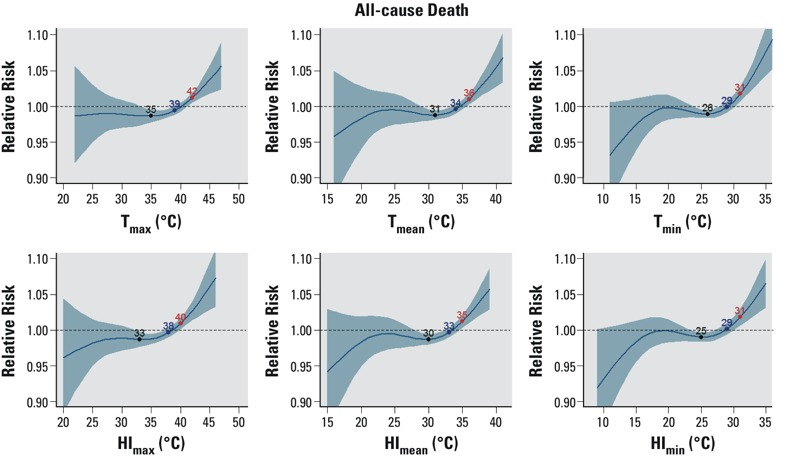
The modeled relationship between the relative risk of all-cause mortality and six different same-day temperature metrics during the warm season for Maricopa County, Arizona, 2000–2011. The solid blue line shows the relative risk of mortality, and the shaded blue region shows the 95% confidence interval. Specific points labeled on the curve identify the minimum risk temperature (MRT, black), the increasing risk temperature (IRT, blue), and the excess risk temperature (ERT, red), representing different conceptualizations of trigger points for intervention activities as discussed in “Methods.”

Across all temperature metrics, the relative risk of all-cause mortality at the highest recorded temperatures exceeded 1.05, with 95% confidence intervals that excluded 1.0 (Figure 1). All three trigger points (MRT, IRT, and ERT) were identified for all six temperature metrics. Regardless of the temperature metric examined, the ERT estimate for all-cause mortality was 2–3°C higher than the IRT, and the IRT estimate was 3–5°C higher than the MRT.

**Figure 2:**
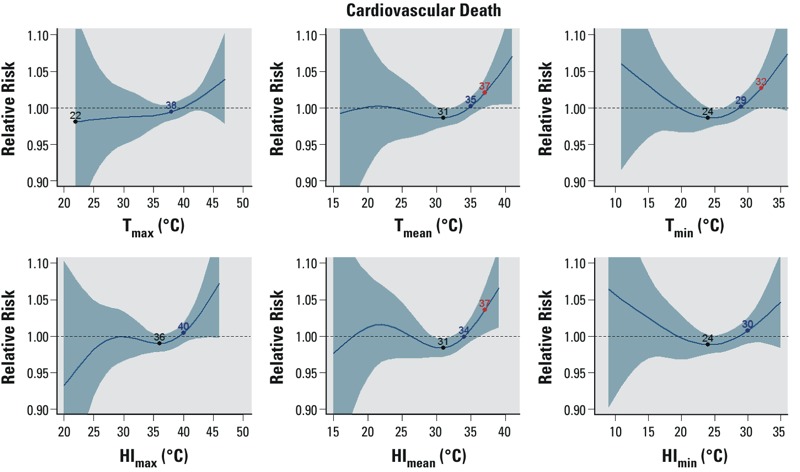
The modeled relationship between the relative risk of cardiovascular mortality and six different temperature metrics with a 1-day lag, as in Figure 1. Fewer than three points are indicated on the curve if some of the trigger points could not be identified.

CVD mortality increased with temperature with a 1-day lag (Figure 2). Relative risks exceeded 1.05 with 95% confidence intervals that excluded 1.0 for some temperature metrics at the highest temperatures. CVD trigger points were less consistent than those for all-cause mortality: an ERT estimate could not be identified for T_max_, HI_max_, and HI_min_, and there was a large difference in IRT and MRT using T_max_ (22 and 36°C, respectively). Where trigger points could be identified, the ERT was 2–3°C higher than the IRT, and, with the exception of T_max_, the IRT was 3–6°C higher than the MRT. The number of CVD deaths (*n* = 30,531) was substantially smaller than the number of deaths from all causes (*n* = 112,853), and the lack of consistency may be a consequence of random error due to the smaller sample size.

No clear pattern of increased risk with higher temperature (1-day lag) emerged for CVD hospitalization or ED visits with CVD listed as the first discharge diagnosis (see Supplemental Material, Figures S2 and S3). Consequently, trigger points could not be identified for these health events for any temperature metric.

**Figure 3:**
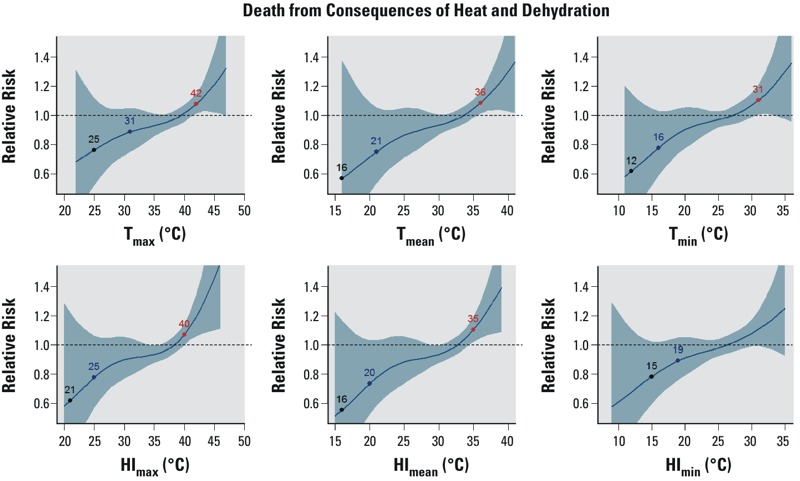
The modeled relationship between the relative risk of mortality from consequences of heat and dehydration and six different temperature metrics with a 1-day lag, as in Figure 1. Fewer than three points are indicated on the curve if some of the trigger points could not be identified.

For the category of conditions called “consequences of heat and dehydration,” the relationship with temperature was consistently positive for mortality, hospitalization, and ED visits (Figure 3; see also Supplemental Material, Figures S4 and S5), but the confidence intervals were wide. The slope of the relationship was shallow. The MRTs and IRTs were much lower for this category of conditions than for all-cause mortality, CVD mortality, and heat-related conditions. For example, considering T_max_, the MRT and IRT were 25°C and 31°C, respectively, for mortality due to conditions considered consequences of heat and dehydration, whereas the MRT and IRT were 35°C and 39°C, respectively, for all-cause mortality.

**Figure 4:**
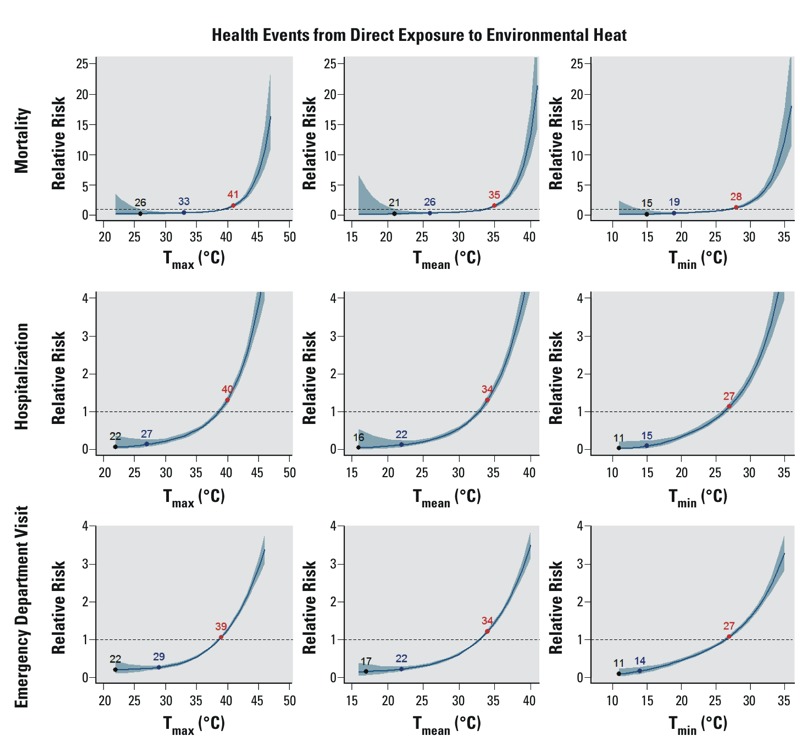
The modeled relationship between the relative risk of heat-related mortality (top panels), heat-related hospitalization (middle panels), and heat-related emergency department visits (lower panels), and three same-day temperature metrics (T_max_, T_mean_, T_min_) during the warm season for Maricopa County, Arizona, 2000–2011 (2008–2012 for morbidity), as in Figure 1. For heat-related events, MRT is the temperature at which the fewest events were observed. Note that the vertical axis scale varies between panels.

**Figure 5:**
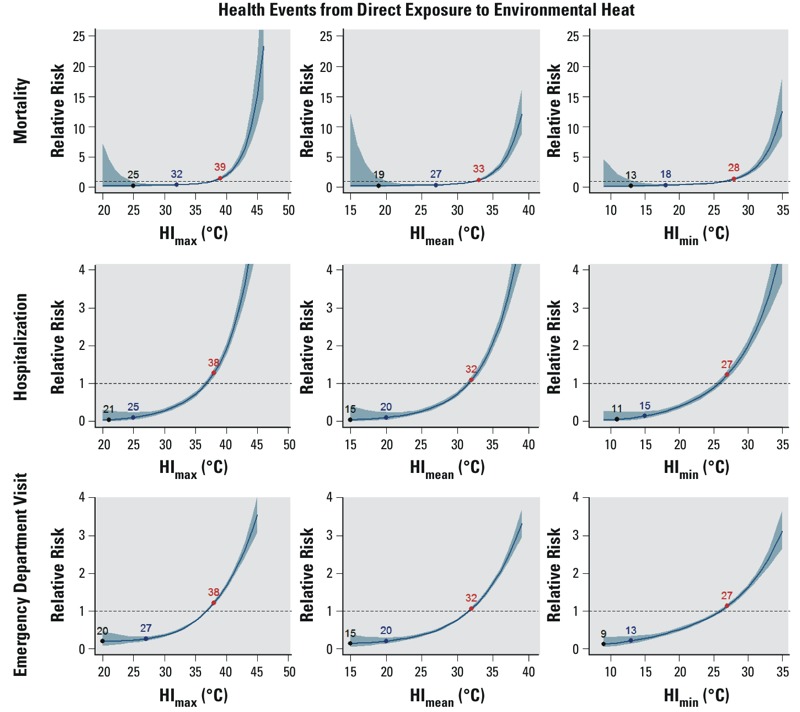
The modeled relationship between the relative risk of heat-related mortality (top panels), heat-related hospitalization (middle panels), and heat-related emergency department visits (lower panels), and three same-day heat index metrics (HI_max_, HI_mean_, HI_min_), as in Figure 1. MRT is the temperature at which the fewest events were observed. Note that the vertical axis scale varies between panels.

We found strong and statistically significant associations between same-day temperature and the three directly heat-related health events (Figures 4 and 5). The relationship exhibited an exponential pattern across all temperature metrics and types of events. MRTs, IRTs, and ERTs were identified for all six temperature metrics for all types of heat-related events. Notably, for all of the temperature metrics, both the MRT and the IRT were consistently 2–7°C lower for heat-related hospitalization and heat-related ED visits than for heat-related mortality. For example, considering T_max_, the corresponding MRT was 26°C for mortality, but 22°C for hospitalization and 22°C for ED visits; similarly, the IRT was 33°C for mortality, but 27°C for hospitalization and 29°C for ED visits. For all of the temperature metrics, however, the ERT was almost the same (± 1–2°C) for each type of heat-related event. For example, considering HI_max_, the ERT was 39°C for heat-related death and 38°C for both hospitalization and ED visits.

**Figure 6:**
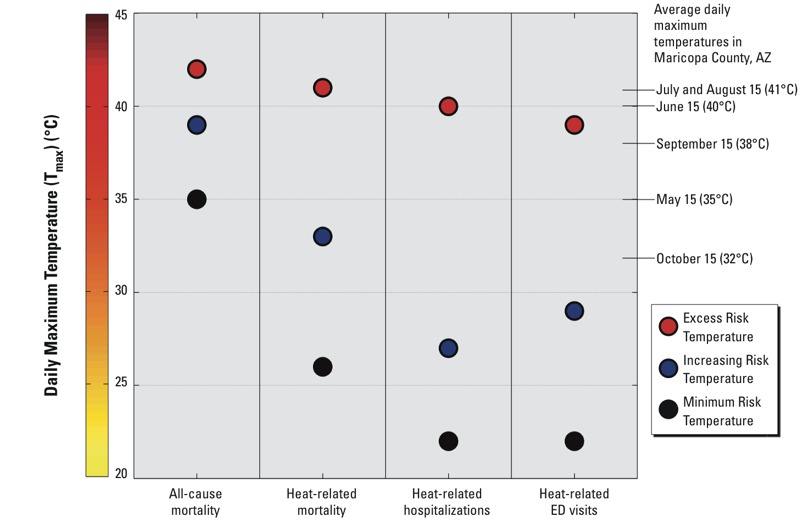
Minimum, increasing, and excess risk temperatures (MRT, IRT, ERT) based on daily maximum temperature (T_max_) for four health events examined in this study. Values on the right-hand side of the figure denote climatological averages at regularly spaced intervals during the warm season in Maricopa County.

The conceptualization of trigger point and choice of health event and diagnosis led to large contrasts in the temperatures at which estimated heat risk increased. Table 2 lists the MRT, IRT, and ERT for 8 of the 10 health events considered in order to facilitate comparisons across categories, event types, temperature metrics, and risk levels; comparisons for T_max_ for select events are also illustrated in Figure 6. Cardiovascular morbidity events are excluded from these tables and figures because of the lack of a consistent association with any temperature metric. Spanning the entire range of risk temperatures, health events, and categories of mortality and morbidity, we observed that trigger points varied by as much as 22°C, holding the temperature metric constant. For example, the ERT for all-cause mortality (considering T_max_) was 42°C, but the MRT for heat-related mortality was 26°C. When examining contrasts across metrics within each type of health event, the MRT, IRT, and ERT were often within 2°C for the air temperature and HI forms of the metric. When the trigger points differed, in most cases, the HI trigger point was 1–2°C lower than the air temperature trigger point.

**Table 2:** Excess, increasing, and minimum risk temperatures in degrees Celsius by category and event type for each temperature metric.

Category/event type	T_max_	HI_max_	T_mean_	HI_mean_	T_min_	HI_min_
Excess risk temperature
Mortality
All-cause	42	40	36	35	31	31
Cardiovascular	—	—	37	37	32	—
Heat-related	41	39	35	33	28	28
Consequences of heat and dehydration	42	40	36	35	31	—
Hospitalization
Heat-related	40	38	34	32	27	27
Consequences of heat and dehydration	42	—	36	35	32	31
ED visits
Heat-related	39	38	34	32	27	27
Consequences of heat and dehydration	40	38	34	33	28	28
Increasing risk temperature
Mortality
All-cause	39	38	34	33	29	29
Cardiovascular	36	40	34	34	29	30
Heat-related	33	32	26	27	19	18
Consequences of heat and dehydration	31	25	21	20	16	19
Hospitalization
Heat-related	27	25	22	20	15	15
Consequences of heat and dehydration	27	26	25	23	21	16
ED visits
Heat-related	29	27	22	20	14	13
Consequences of heat and dehydration	31	23	20	18	14	11
Minimum risk temperature
Mortality
All-cause	35	33	31	30	26	25
Cardiovascular	22	36	31	31	24	24
Heat-related	26	25	21	19	15	13
Consequences of heat and dehydration	25	21	16	16	12	15
Hospitalization
Heat-related	22	21	16	15	11	11
Consequences of heat and dehydration	22	20	17	17	14	9
ED visits
Heat-related	22	20	17	15	11	9
Consequences of heat and dehydration	29	20	17	16	11	9

Sensitivity analyses revealed that the overall scale and pattern of the differences between trigger points based on different conceptualizations of thresholds was consistent regardless of the specific time period examined, although the specific values of the MRT, IRT, and ERT were not identical for all examined time periods (see Supplemental Material, Tables S4 and S5).

## Discussion

Most prior analyses of temperature/event associations that aim to identify a threshold temperature for heat-related events, including our own work set in Maricopa County (Harlan et al. 2014), define the threshold for action as the temperature at which the frequency of health events begins to rise rapidly (most similar to the ERT in this analysis for all-cause mortality, CVD mortality, and heat-related events) although other definitions have been used (e.g., Armstrong et al. 2011; Hajat and Kosatsky 2010; Loughnan et al. 2010; Zaninović and Matzarakis 2014). A statistically solid and reliable health outcomes–based estimate of temperature trigger points has the potential to guide the implementation of interventions when they are most appropriate. Issuing extreme heat warning products to the general public by weather forecasting offices is one such intervention (e.g., Pascal et al. 2006; Williams et al. 2012a), but triggering criteria for warning systems are often based on threshold conditions for a singular conceptualization of increases in all-cause mortality (e.g., Hondula et al. 2014). An understanding of the broader effects of heat on illness has the potential to suggest enhancements to public messaging efforts as well as interventions other than warnings that might mitigate the adverse effects of heat.

Here, we have interrogated temperature threshold estimates based on three different criteria (MRT, IRT, and ERT). We found large differences across these measures and across different health events and diagnoses. The strongest and most consistent associations for high environmental temperature in our setting were with directly heat-related health events. Trigger points for these events were consistently lower than those derived from all-cause mortality. In a hot location like Maricopa County, using a single high threshold temperature (e.g., ERT for all-cause mortality) vastly discounts the number of days on which heat is associated with an increased risk of heat-related mortality and morbidity. This progression of increasing thresholds for more severe outcomes and the overall finding that heat-related mortality is merely the top of the heat severity pyramid was also reported in Adelaide, South Australia (Williams et al. 2012a). The highest trigger points (ERTs) that we calculated for several health events were near climatological averages for summer daily temperatures (Figure 6). This finding demonstrates a need to reconsider the heat-risk communication paradigm in hot climates. We suggest that one improvement would be for researchers to offer intended end-users an array of trigger points that could be applied for their specific purposes instead of a single, all-purpose threshold temperature. In Maricopa County, we are using the results of this study to begin conversations with a range of end-users about actions they could take when dangerous heat occurs. The ultimate utility of the trigger points will be determined after engaging in dialogue with service providers. Potential applications for these trigger points include identifying days and times to increase enforcement of workplace safety guidelines, running seasonal public awareness campaigns, suspending utility shutoffs, rescheduling or cancelling outdoor school events including athletic practices and competitions, and opening or expanding access to homeless shelters and cooling centers. The trigger point framework may also offer additional opportunities to consider multiple health outcomes, risk levels, and exposure variables in studies that project future heat impacts associated with climate change.

The HI, which is widely used by the NWS and heat-health researchers in the United States (e.g., Anderson et al. 2013), provided information about sensitivity to heat that was not substantively different from information derived from air temperature in Maricopa County. In our study setting, and perhaps in others characterized by low relative humidity, actions to mitigate the effects of heat on health events may not need to use metrics that are more complex than air temperature and are, therefore, more difficult to communicate to the public. Identification of the optimal variable(s) to use when triggering protective actions related to extreme heat depends on rigorous statistical analysis of predictive capacity (e.g., Barnett et al. 2010; Zhang et al. 2012), local context, and public understanding of and receptivity to such information. Exploration of these important dimensions of heat intervention design falls outside the scope of this analysis but is the subject of ongoing efforts by the authors and local public agencies.

Notably, our study did not find an association between high temperatures and CVD hospitalization and/or ED visits (see Supplemental Material, Figures S2 and S3). In a recent systematic review of studies of heat and cardiovascular morbidity, Turner et al. (2012) concluded that the effects of temperature on cardiorespiratory morbidity were smaller and more variable than those on mortality. Administrative data have a limited ability to shed light on the effects of temperature on CVD morbidity. As others have noted (Basu et al. 2012), more studies that assess specific symptoms in relation to individual heat exposure are needed.

Our study has several important limitations. We used administrative data to assess hospitalization and ED visits, as has been done in previous studies (e.g., Williams et al. 2012b), although the data sets were created to support insurance billing and not for use in this type of research. Our methodology of using ICD-10 codes to identify heat-related mortality from ADHS records underestimates the number of heat deaths. In particular, Maricopa County’s procedures to identify heat-related deaths have been improving over time, and their heat mortality surveillance program detected 312 heat-related deaths during the period 2008–2011 [Maricopa County Department of Public Health (MCDPH) 2014] compared with the 153 heat-related deaths that we identified using procedures more consistent with those employed by ADHS.

It is also worth noting that our study focused on a single setting; thus, our findings may not be generalizable to other settings. There are many human adaptations to high temperatures, and Maricopa County may be particularly heat-adapted (Hartz et al. 2013). Because the presence of dangerously hot weather in the summer is predictable in this setting, some residents travel to cooler places and may be able to avoid activities that involve heat exposure. During the study period, heat warnings, networks for water distribution, and cooling facilities were available to the public. These efforts may have mitigated the effects of heat on illness and death. There are potential modifiers of the temperature-health relationship that we did not examine, including air pollution, time of season, cumulative days of high temperatures, and displacement. The applications of this framework should be updated continually. Trigger points should be monitored and evaluated for changes because of temporal variability in weather and climate [indicated by the reevaluation of climate “normals,” Arguez et al. (2012)] and because the behavior of people, the physical environment (e.g., building materials), the availability of technology (e.g., air conditioning), and public health systems adapt to higher temperatures in ways that may affect the human health response to heat (Guo et al. 2012). Finally, the meteorological data were obtained from a single station, whereas the health events were experienced across a larger geographic area.

## Conclusion

In summary, this study found strong and consistent associations of environmental temperature with all-cause mortality, CVD mortality, heat-related mortality, hospitalization, and ED visits and with a category of conditions considered possible consequences of heat or dehydration based on pathophysiologic reasoning. Consideration of different health events and various conceptualizations of threshold temperatures revealed large contrasts in the trigger points at which activation of different heat intervention efforts might be appropriate. Plans to mitigate the effects of high environmental heat on human health that incorporate different levels of sensitivity for determining the most effective adaptation strategies and when to deploy them might have important benefits in terms of illnesses and deaths avoided.

## Supplemental Material

(1.2 MB) PDF
