# Supplementary material for: Multiple Trigger Points for Quantifying Heat-Health Impacts: New Evidence from a Hot Climate
Source: Environ Health Perspect. 2015 Jul 28;124(2):176–83. doi: 10.1289/ehp.1409119 (PMC4749077; doi:10.1289/ehp.1409119)
Supplement: (1.2 MB) PDF [file ehp.1409119.s001.acco.pdf]

Environ Health Perspect
DOI: 10.1289/ehp.1409119

Note to Readers: *EHP* strives to ensure that all journal content is accessible to all readers. However, some figures and Supplemental Material published in *EHP* articles may not conform to 508 standards due to the complexity of the information being presented. If you need assistance accessing journal content, please contact ehp508@niehs.nih.gov. Our staff will work with you to assess and meet your accessibility needs within 3 working days.

Supplemental Material

Multiple Trigger Points for Quantifying Heat-Health Impacts: New Evidence from a Hot Climate

Diana B. Petitti, David M. Hondula, Shuo Yang, Sharon L. Harlan, and Gerardo Chowell

Table of Contents

Table S1. Conditions Used to Define Cardiovascular Disease Events and Corresponding ICD-10 and ICD-9-CM Codes

Table S2. Conditions Used to Define Heat-Related Events and Corresponding ICD-10 and ICD-9-CM Codes

Table S3. Conditions Used to Define Category of Events: Possible Consequences of Heat or Dehydration and Corresponding ICD-10 and ICD-9-CM Codes

Algorithm for Calculation of Heat Index Based on Steadman 1979; NWS 2014

Figure S1. Framework for Physiologic and Pathophysiologic Effects of Heat or Dehydration on Conditions Used to Define This Category

Figure S2. Modeled Relationship Between Relative Risk of Cardiovascular Hospitalizations and Six Different Temperature Metrics

Figure S3. Modeled Relationship Between Relative Risk of Cardiovascular Emergency Department Visits and Six Different Temperature Metrics. First Diagnosis Only

Figure S4. Modeled Relationship Between Relative Risk of Consequences of Heat and Dehydration Hospitalizations and Six Different Temperature Metrics

Figure S5. Modeled Relationship Between Relative Risk of Consequences of Heat and Dehydration Emergency Department Visits and Six Different Temperature Metrics.

Tables S4 and S5. The tables show the results of sensitivity analyses in which the Minimum, Increasing, and Excess Risk Temperatures (MRT, IRT, ERT, respectively) were calculated using different time periods for the health and meteorological data. The values in the table compare trigger points for the four health events emphasized in this manuscript and are based on daily maximum temperature. An asterisk (*) indicates the time period examined in the main text. All values shown are in degrees Celsius (°C).

References

Table S1. Conditions Used to Define Cardiovascular Disease Events and Corresponding ICD-10 and ICD-9-CM Codes

**Condition Description**	**ICD 10 Code**	**ICD-9-CM Code**
Unstable angina	I20.0	411.1
Angina pectoris with documented spasm	I20.1	413.1
Other forms of angina pectoris	I20.8	413.9
Angina pectoris, unspecified	I20.9	413.9
Acute transmural myocardial infarction of anterior wall	I21.0	410.11
Acute transmural myocardial infarction of inferior wall	I21.1	410.41
Acute transmural myocardial infarction of other sites	I21.2	410.81
Acute transmural myocardial infarction of unspecified site	I21.3	410.91
Acute subendocardial myocardial infarction	I21.4	410.71
Acute myocardial infarction, unspecified	I21.9	410.91
Subsequent myocardial infarction of anterior wall	I22.0	410.11
Subsequent myocardial infarction of inferior wall	I22.1	410.41
Subsequent myocardial infarction of other sites	I22.8	410.81
Subsequent myocardial infarction of unspecified site	I22.9	410.91
Dressler's syndrome	I24.1	411.0
Other forms of acute ischemic heart disease	I24.8	411.89
Acute ischemic heart disease, unspecified	I24.9	411.89
Atherosclerotic heart disease	I25.1	414.00
Old myocardial infarction	I25.2	412
Aneurysm of heart	I25.3	414.19
Coronary artery aneurysm	I25.4	414.11
Ischemic cardiomyopathy	I25.5	414.8
Silent myocardial ischemia	I25.6	414.8
Other forms of chronic ischemic heart disease	I25.8	414.8
Chronic ischemic heart disease, unspecified	I25.9	414.9
Cardiac arrhythmia, unspecified	I49.9	427.9
Atherosclerotic cardiovascular disease, so described	I25.0	429.2
Subarachnoid hemorrhage from carotid siphon and bifurcation	I60.0	430
Subarachnoid hemorrhage from middle cerebral artery	I60.1	430
Subarachnoid hemorrhage from anterior communicating artery	I60.2	430
Subarachnoid hemorrhage from posterior communicating artery	I60.3	430
Subarachnoid hemorrhage from basilar artery	I60.4	430
Subarachnoid hemorrhage from vertebral artery	I60.5	430
Subarachnoid hemorrhage from other intracranial arteries	I60.6	430

Subarachnoid hemorrhage from intracranial artery, unspecified	I60.7	430
Other subarachnoid hemorrhage	I60.8	430
Subarachnoid hemorrhage, unspecified	I60.9	430
Intracerebral hemorrhage in hemisphere, subcortical	I61.0	431
Intracerebral hemorrhage in hemisphere, cortical	I61.1	431
Intracerebral hemorrhage in hemisphere, unspecified	I61.2	431
Intracerebral hemorrhage in brain stem	I61.3	431
Intracerebral hemorrhage in cerebellum	I61.4	431
Intracerebral hemorrhage, intraventricular	I61.5	431
Intracerebral hemorrhage, multiple localized	I61.6	431
Other intracerebral hemorrhage	I61.8	431
Intracerebral hemorrhage, unspecified	I61.9	431
Nontraumatic extradural hemorrhage	I62.1	432.0
Subdural hemorrhage (acute) (nontraumatic)	I62.0	432.1
Intracranial hemorrhage (nontraumatic), unspecified	I62.9	432.9
Cerebral infarction due to thrombosis of precerebral arteries	I63.0	433.91
Cerebral infarction due to embolism of precerebral arteries	I63.1	433.91
Cerebral infarction due to unspecified occlusion or stenosis of precerebral arteries	I63.2	433.91
Cerebral infarction due to thrombosis of cerebral arteries	I63.3	434.01
Cerebral infarction due to embolism of cerebral arteries	I63.4	434.11
Cerebral infarction due to unspecified occlusion or stenosis of cerebral arteries	I63.5	434.91
Other cerebral infarction	I63.8	434.91
Cerebral infarction, unspecified	I63.9	434.91
Stroke, not specified as hemorrhage or infarction	I64.	436
Cerebral atherosclerosis	I67.2	437.0
Other specified cerebrovascular diseases	I67.8	437.1
Hypertensive encephalopathy	I67.4	437.2
Cerebral aneurysm, nonruptured	I67.1	437.3
Cerebral arteritis, not elsewhere classified	I67.7	437.4
Moyamoya disease	I67.5	437.5
Cerebral infarction due to cerebral venous thrombosis, nonpyogenic	I63.6	437.6
Nonpyogenic thrombosis of intracranial venous system	I67.6	437.6
Dissection of cerebral arteries, nonruptured	I67.0	437.9
Cerebrovascular disease, unspecified	I67.9	437.9
Sequelae of subarachnoid hemorrhage	I69.0	438
Sequelae of intracerebral hemorrhage	I69.1	438
Sequelae of other nontraumatic intracranial hemorrhage	I69.2	438
Sequelae of cerebral infarction	I69.3	438
Sequelae of stroke, not specified as hemorrhage or infarction	I69.4	438

Sequelae of other and unspecified cerebrovascular diseases	I69.8	438
Atherosclerosis of aorta	I70.0	440.0
Atherosclerosis of renal artery	I70.1	440.1
Atherosclerosis of arteries of the extremities	I70.2	440.20
Atherosclerosis of other arteries	I70.8	440.8
Generalized and unspecified atherosclerosis	I70.9	440.9
Congestive heart failure	I50.0	428.0
Left ventricular failure	I50.1	428.1
Heart failure, unspecified	I50.9	428.9
Cardiovascular disease, unspecified	I51.6	429.2
Other ill-defined heart diseases	I51.8	429.89
Heart disease, unspecified	I51.9	429.9

Table S2. Conditions Used to Define Heat-Related Events and Corresponding ICD-10 and ICD-9-CM Codes

Condition Description	ICD 10 Code	ICD-9-CM Code
Sunburn of third degree	L55.2	692.71
Other sunburn	L55.8	692.71
Sunburn, unspecified	L55.9	692.71
Drug phototoxic response	L56.0	692.72
Drug photoallergic response	L56.1	692.72
Photocontact dermatitis [berloque dermatitis]	L56.2	692.72
Polymorphous light eruption	L56.4	692.72
Other specified acute skin changes due to ultraviolet radiation	L56.8	692.72
Acute skin change due to ultraviolet radiation, unspecified	L56.9	692.79A
Solar urticarial	L56.3	708.8
Heatstroke and sunstroke	T67.0	992.0
Heat syncope	T67.1	992.1
Heat cramp	T67.2	992.2
Heat exhaustion, anhidrotic	T67.3	992.3
Heat exhaustion due to salt depletion	T67.4	992.4
Heat exhaustion, unspecified	T67.5	992.5
Heat fatigue, transient	T67.6	992.6
Heat oedema	T67.7	992.7
Other effects of heat and light	T67.8	992.8
Effect of heat and light, unspecified	T67.9	992.9
Exposure to excessive natural heat	X30	E900.00
Exposure to sunlight	X32	E900.00

Keywords/Text String for Deaths: Environm#, Environmental Heat, Excessive Heat, Excessive Natural Heat, Exposure to Heated Environment, Exposure to High Environmental Temperature, Exposure to Hot Desert Environment, Exposure to Hot Environment, Heat Cramps, Heat Effect, Heat Environment, Heat Exhaustion, Heated Environment, Heat Exposure, Heat Related, Heat Stress, Heat Syncope, High Environmental Temperature, Hiking in Hot Climate, Temperature, Sun

Table S3. Conditions Used to Define Category of Events: Possible Consequences of Heat or Dehydration and Corresponding ICD-10 and ICD-9-CM Codes

Condition Description	ICD 10 Code	ICD-9-CM Code
Hypo-osmolality and hyponatremia	E87.1	276.1
Mixed disorder of acid-base balance	E87.4	276.4
Hyperkalemia	E87.5	276.7
Hypokalemia	E87.6	276.8
Disseminated intravascular coagulation [defibrination syndrome]	D65	286.6
Supraventricular tachycardia	I47.1	427.0
Ventricular tachycardia	I47.2	427.1
Paroxysmal tachycardia, unspecified	I47.9	427.2
Atrial fibrillation and flutter	I48	427.31
Ventricular fibrillation and flutter	I49.0	427.41
Cardiac arrest with successful resuscitation	I46.0	427.5
Sudden cardiac death, so described	I46.1	427.5
Cardiac arrest, unspecified	I46.9	427.5
Atrial premature depolarization	I49.1	427.61
Junctional premature depolarization	I49.2	427.69
Ventricular premature depolarization	I49.3	427.69
Other and unspecified premature depolarization	I49.4	427.69
Sick sinus syndrome	I49.5	427.81
Reentry ventricular arrhythmia	I47.0	427.89
Other specified cardiac arrhythmias	I49.8	427.89
Bradycardia, unspecified	R001	427.89
Orthostatic hypotension	I95.1	458.0
Other hypotension	I95.8	458.1
Idiopathic hypotension	I95.0	458.9
Hypotension, unspecified	I95.9	458.9
Acute renal failure with tubular necrosis	N17.0	584.5
Acute renal failure with acute cortical necrosis	N17.1	584.6
Acute renal failure with medullary necrosis	N17.2	584.7
Other acute renal failure	N17.8	584.8
Acute renal failure, unspecified	N17.9	584.9
Calculus of kidney	N20.0	592.0
Calculus of kidney with calculus of ureter	N20.2	592.0
Calculus of ureter	N20.1	592.1
Urinary calculus, unspecified	N20.9	592.9

Calculus in bladder	N21.0	594.0
Calculus in urethra	N21.1	594.2
Other lower urinary tract calculus	N21.8	594.8
Calculus of lower urinary tract, unspecified	N21.9	594.9
Disturbances of sodium balance of newborn	P742	775.5
Disturbances of potassium balance of newborn	P743	775.5
Environmental hyperthermia of newborn	P810	778.4
Coma, unspecified	R40.2	780.01
Syncope and collapse	R55	780.2
Febrile convulsions	R56.0	780.31
Fever, unspecified	R50.9	780.6
Malaise and fatigue	R53	780.7
Tachycardia, unspecified	R00.0	785.0
Palpitations	R00.2	785.1
Shock, unspecified	R57.9	785.50
Hypovolemic shock	R57.1	785.59
Other shock	R57.8	785.59
Other and unspecified abnormalities of heart beat	R00.8	785.9
Unspecified renal colic	N23	788.0
Instantaneous death	R96.0	798.1
Death occurring less than 24 hours from onset of symptoms, not otherwise explained	R96.1	798.2
Unattended death	R98	798.9

Keywords/Text String for Deaths: Exhaustion, Hyperkalemia, Hypernatremia, Hyperther, Hyperthermia, Hypokalemia, Hyponatremia, Hypovolemia

Algorithm for Calculation of Heat Index Based on Steadman 1979; NWS 2014

The equation used to calculate the heat index *(HI)* is:

$$HI = -42.379 + 2.04901523 \times T + 10.14333127 \times RH - 0.22475541 \times T \times RH - 0.00683783 \times T^2$$
$$-0.05481717 \times RH^2 + 0.00122874 \times T^2 \times RH + 0.00085282 \times T \times RH^2 - 0.00000199 \times T^2 \times RH^2$$

[S1]

where *T* is temperature in °F and *RH* is relative humidity in percent.

If the ***RH*** is less than 13% and the temperature is between 80 and 112 °F, then the following adjustment value is subtracted from *HI*:

$$Adjustment = [(13 - RH)/4] \times \sqrt{[(17 - |T - 95|)/17]} \qquad [S2]$$

If the *RH* is greater than 85% and the temperature is between 80 and 87°F, then the following adjustment value is added to *HI*:

$$Adjustment = [(RH - 85)/10] \times [(87 - T)/5] \qquad [S3]$$

When meteorological conditions result in a heat index of 80°F or below, a separate equation is used:

$$HI = 0.5 \times (T + 61.0 + [(T - 68.0) \times 1.2] + (RH \times 0.994)) \qquad [S4]$$

Figure S1. Framework for Physiologic and Pathophysiologic Effects of Heat or Dehydration on Conditions Used to Define This Category

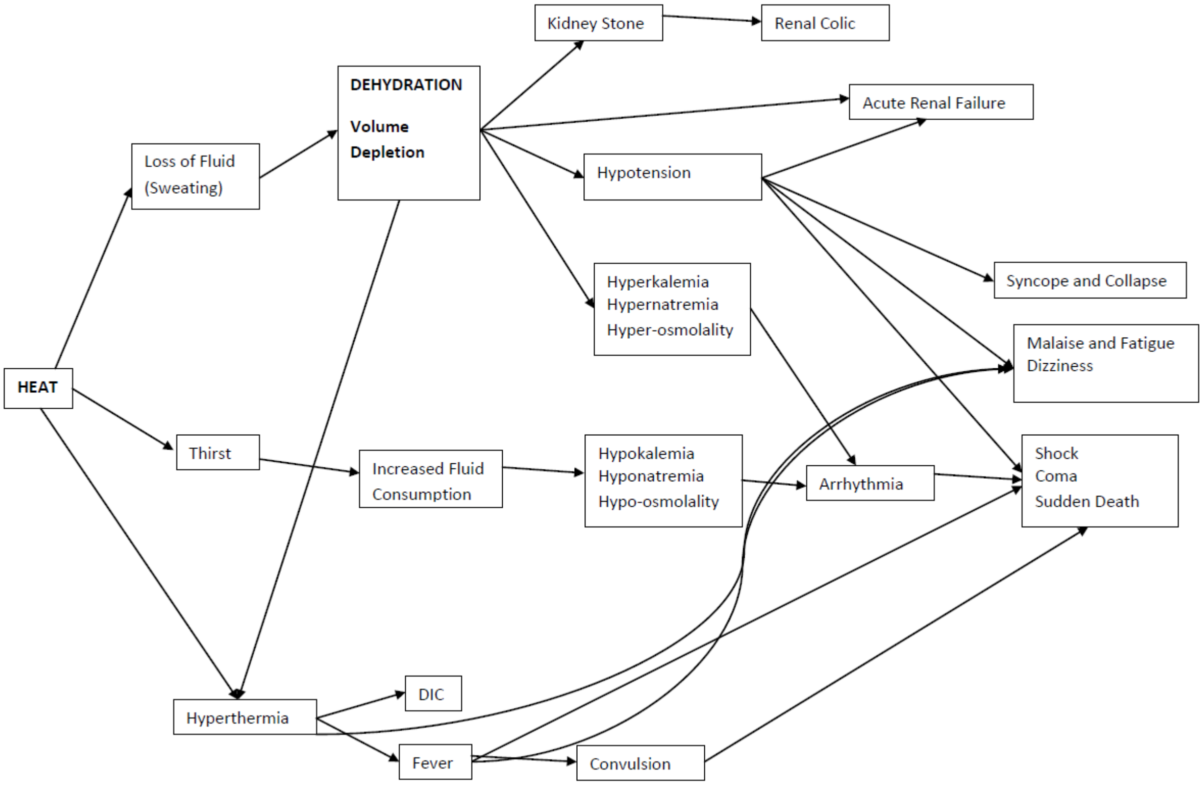

Legend: Physiologic / pathophysiologic framework for selection of conditions considered to consequences of heat and dehydration.

Figure S2. Modeled Relationship Between Relative Risk of Cardiovascular Hospitalizations and Six Different Temperature Metrics.

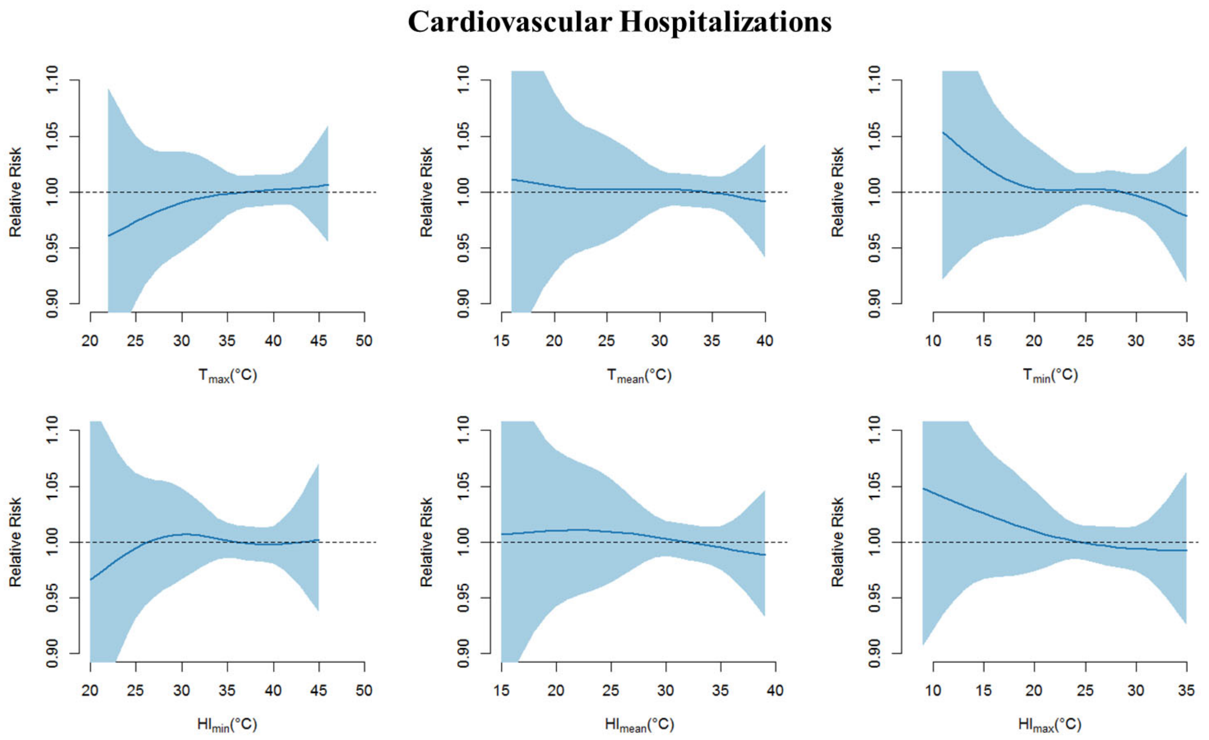

Legend. The solid blue line shows the relative risk of cardiovascular hospitalizations and the shaded blue region shows the 95% confidence interval. No trigger points could be identified for any of the temperature metrics considered. Note: A one-day lag is used for all temperature metrics for estimating effects on cardiovascular hospitalizations.

Figure S3. Modeled Relationship Between Relative Risk of Cardiovascular Emergency Department Visits and Six Different Temperature Metrics. First Diagnosis Only

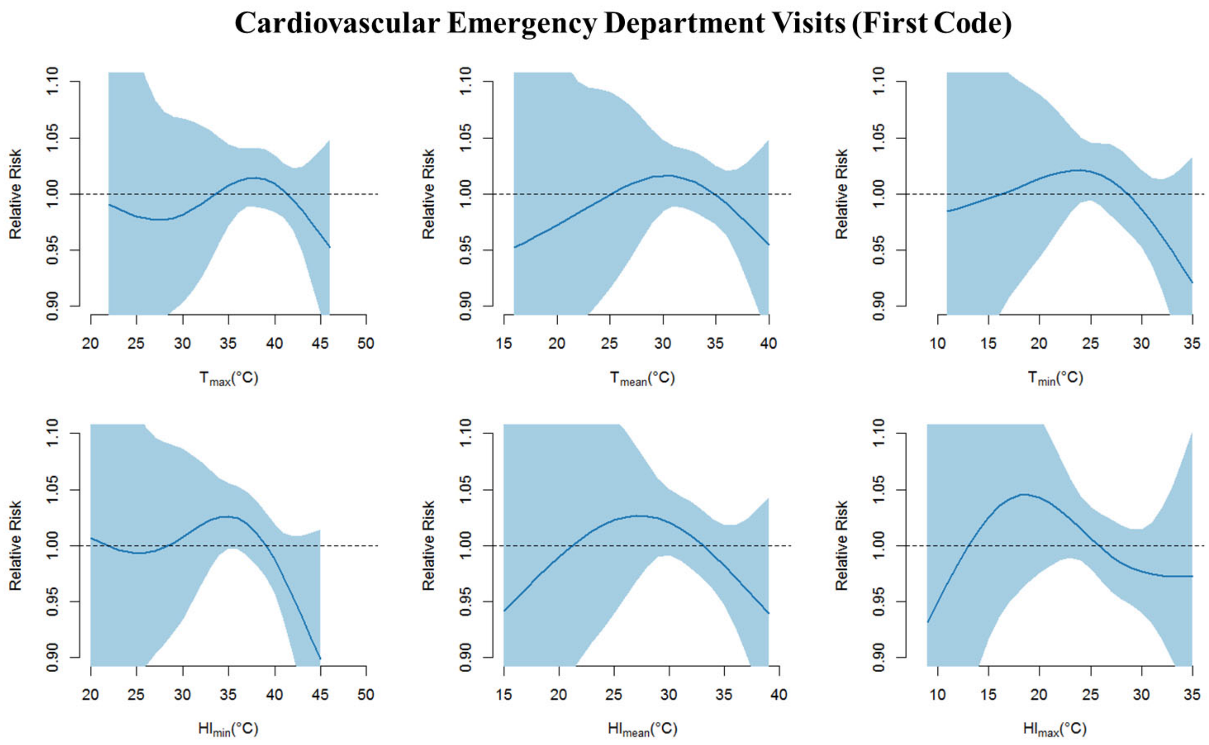

Legend. The solid blue line shows the relative risk of cardiovascular emergency department visits and the shaded blue region shows the 95% confidence interval. No trigger points could be identified for any of the temperature metrics considered. Note: A one-day lag is used for all temperature metrics for estimating effects on cardiovascular emergency department visits.

Figure S4. Modeled Relationship Between Relative Risk of Consequences of Heat and Dehydration Hospitalizations and Six Different Temperature Metrics.

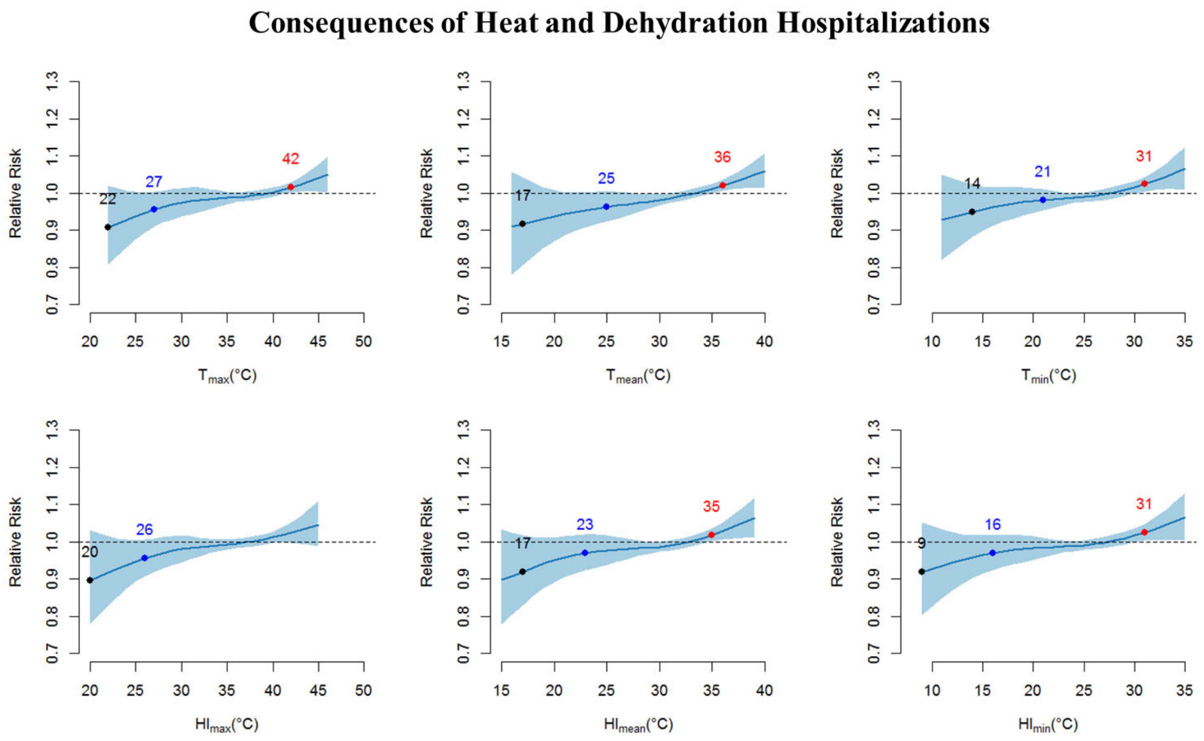

Legend. The solid blue line shows the relative risk of hospitalizations for consequences of heat and dehydration and the shaded blue region shows the 95% confidence interval. Specific points labeled on the curve identify the Minimum Risk Temperature (MRT, black), Increasing Risk Temperature (IRT, blue), and Excess Risk intervention activities as discussed in the Methods section. No ERT could be identified for maximum heat index.

Figure S5. Modeled Relationship Between Relative Risk of Consequences of Heat and Dehydration Emergency Department Visits and Six Different Temperature Metrics.

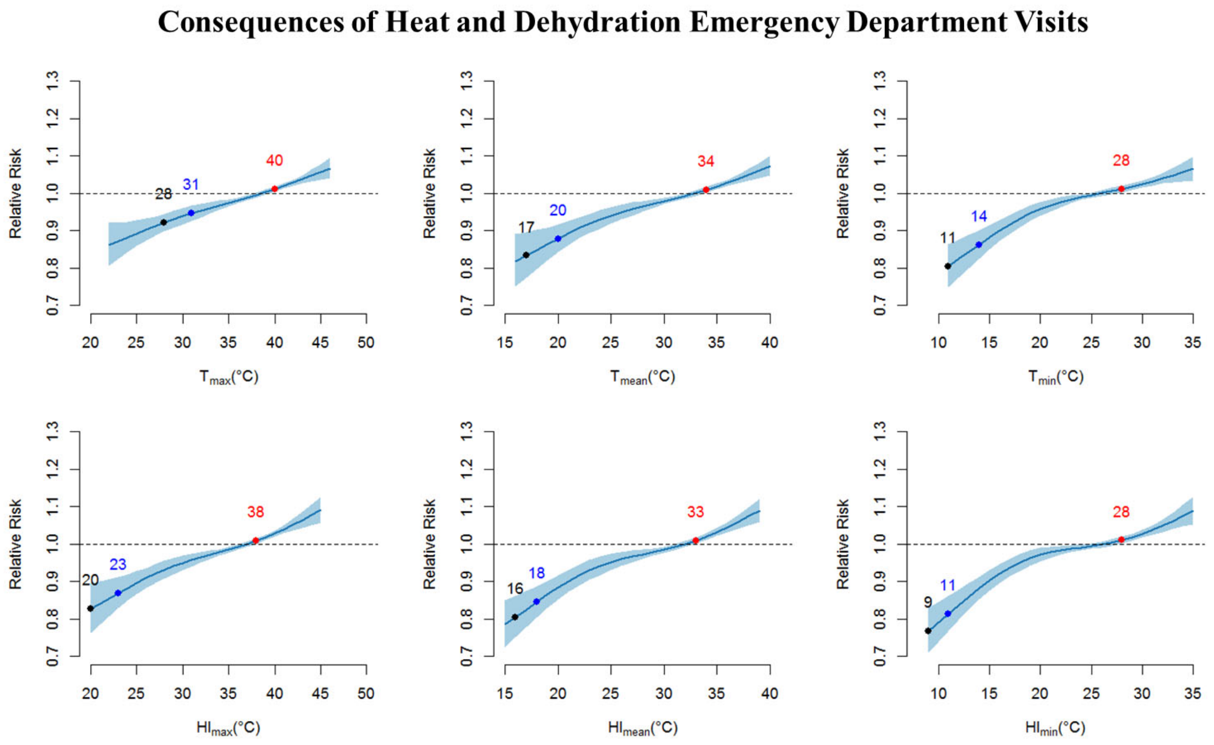

Legend. The solid blue line shows the relative risk of consequences of heat and dehydration emergency department visits and the shaded blue region shows the 95% confidence interval. Specific points labeled on the curve identify the Minimum Risk Temperature (MRT, black), Increasing Risk Temperature (IRT, blue), and Excess Risk Temperature (ERT, red), representing different conceptualizations of trigger points for intervention activities as discussed in the Methods section.

Tables S4 and S5. The tables show the results of sensitivity analyses in which the Minimum, Increasing, and Excess Risk Temperatures (MRT, IRT, ERT, respectively) were calculated using different time periods for the health and meteorological data. The values in the table compare trigger points for the four health events emphasized in this manuscript and are based on daily maximum temperature. An asterisk (*) indicates the time period examined in the main text. All values shown are in degrees Celsius (°C).

Table S4 for mortality events:

	All-cause mortality			Heat-related mortality		
Time period	MRT	IRT	ERT	MRT	IRT	ERT
2000–2011*	35	39	42	26	33	41
2002–2011	32	37	41	26	31	40
2004–2011	32	36	41	26	33	41
2006–2011	33	37	41	26	34	41
2000–2009	37	40	42	28	32	41
2000–2007	37	41	43	28	35	41
2000–2005	38	41	43	28	36	41

Table S5 for morbidity events

	Heat-related hospitalizations			Heat-related ED visits		
Time period	MRT	IRT	ERT	MRT	IRT	ERT
2008–2012*	22	27	40	22	29	39
2009–2012	26	30	40	24	30	40
2010–2012	27	30	40	24	30	40
2008–2011	22	27	40	22	30	39
2008–2010	22	27	40	22	30	39

References

Steadman R. 1979. The assessment of sultriness. Part I: A temperature-humidity index based on human physiology and clothing science. J Appl Meteor 18:861-873.

NWS (U.S. National Weather Service). The Heat Index Equation. 2014. Available: http://www.hpc.ncep.noaa.gov/html/heatindex_equation.shtml [accessed 15 May 2014].
